# Computational and Experimental Investigation of the Selective Adsorption of Indium/Iron Ions by the Epigallocatechin Gallate Monomer

**DOI:** 10.3390/ma15228251

**Published:** 2022-11-21

**Authors:** Zhigao Liu, Zhongmin Wang, Weijiang Gan, Songlin Liu, Jianglin Zhang, Zhaojin Ran, Chenxi Wu, Chaohao Hu, Dianhui Wang, Tao Chen, Guiyin Li

**Affiliations:** 1 Guangxi Academy of Sciences, Nanning 530007, China; 2 School of Materials Science and Engineering, Guilin University of Electronic Technology, Guilin 541004, China; 3 College of Chemistry, Guangdong University of Petrochemical Technology, Maoming 525000, China

**Keywords:** epigallocatechin gallate, selective adsorption, indium/iron ions, first-principles calculations

## Abstract

Selective recovery of indium has been widely studied to improve the resource efficiency of critical metals. However, the interaction and selective adsorption mechanism of indium/iron ions with tannin-based adsorbents is still unclear and hinders further optimization of their selective adsorption performance. In this study, the epigallocatechin gallate (EGCG) monomer, which is the key functional unit of persimmon tannin, was chosen to explore the ability and mechanism of selective separation/extraction of indium from indium–iron mixture solutions. The density functional theory calculation results indicated that the deprotonated EGCG was easier to combine with indium/iron cations than those of un-deprotonated EGCG. Moreover, the interaction of the EGCG–Fe(III) complex was dominated by chelation and electrostatic interaction, while that of the EGCG–In(III) complex was controlled by electrostatic interactions and aromatic ring stacking effects. Furthermore, the calculation of binding energy verified that EGCG exhibited a stronger affinity for Fe(III) than that for In(III) and preferentially adsorbed iron ions in acidic or neutral solutions. Further experimental results were consistent with the theoretical study, which showed that the Freundlich equilibrium isotherm fit the In(III) and Fe(III) adsorption behavior very well, and the Fe(III) adsorption processes followed a pseudo-second-order model. Thermodynamics data revealed that the adsorption of In(III) and Fe(III) onto EGCG was feasible, spontaneous, and endothermic. The adsorption rate of the EGCG monomer for Fe(III) in neutral solution (1:1 mixed solution, pH = 3.0) was 45.7%, 4.3 times that of In(III) (10.7%). This study provides an in-depth understanding of the relationship between the structure of EGCG and the selective adsorption capacity at the molecular level and provides theoretical guidance for further optimization of the selective adsorption performance of structurally similar tannin-based adsorbents.

## 1. Introduction

Indium is a rare metal that is widely used in the electronics industry, machinery manufacturing, and other fields owing to its excellent physical, chemical, and optoelectronic properties [1,2]. Indium is sparsely distributed in its ore, and it mainly co-exists with elements such as tin, iron, and zinc, which have properties similar to those of indium itself [3,4]. High-purity indium for industrial applications is extracted from indium-rich ores or recycled from secondary indium sources such as production scrap and end-of-life consumer goods [5,6]. However, the presence of highly-concentrated iron in secondary (waste) sources renders the separation of indium from its ore difficult due to their similar physical properties. Selective recovery of valuable indium metal from secondary sources is critical to improving resource sustainability. Indium-iron separation has therefore become an important step in the production and recovery of high-purity indium [7,8]. Currently, the methods for indium–iron separation include extraction resin separation [9], electrochemical depositions [10,11], biosorption [12,13], and solvent extraction [14,15]. Of these, the common method is solvent extraction, but this process is very easy to co-extract with iron in the process of extracting indium and it is difficult to back extract. However, the selective adsorption method can remove iron impurities and leave indium in the aqueous solution to purify indium ions. Particularly, the adsorption method is considered to be particularly promising for metal recovery due to its simplicity, low cost, and relatively high efficiency.

**Figure 1:**
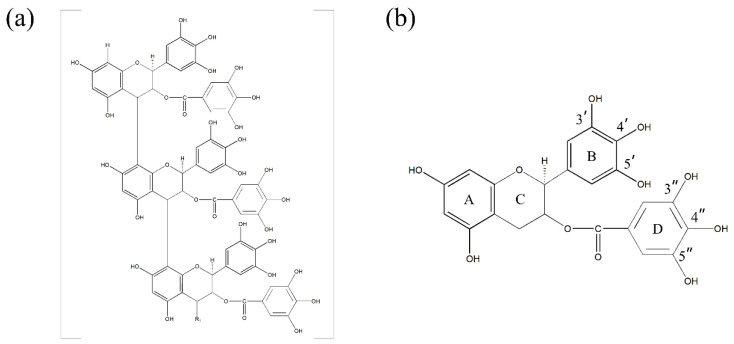
Chemical structures of (**a**) PT and (**b**) EGCG.

Tannin-based adsorbents have a natural affinity to absorb heavy metals and selectively recover precious and critical metals from wastewater [16]. Particularly, persimmon tannin (PT) is a natural polyphenol compound in persimmon and a valuable waste material commonly discarded from the juice industry. Due to the presence of a large number of phenolic hydroxyl groups in the structure of PT, it can efficiently adsorb and selectively bind a variety of metal ions [17]. Li et al. [18] reported that PT has a distinct structure, different from those of other condensation tannins, mainly comprising epicatechin (EC), epigallocatechin (EGC), epicatechin gallate (ECG), and epigallocatechin gallate (EGCG). Importantly, the adsorptive capacity of PT is mainly attributed to EGCG. The structural models of PT and EGCG are shown in Figure 1.

Many studies on the selective adsorption of PTs have been conducted to date [16,19]. Zhang et al. used 2,5-dimercapto-1,3,4-thiadiazole-immobilized persimmon tannin-based bio-sorbent to selectively adsorb Ag(I) from e-waste leachate in the absence of chloride ions. They reported that the modified PT exhibited high adsorption for Ag(I) and almost no adsorption activity for cations such as Zn(II), Pb(II), Cu(II), and Ni(II) in the solution [20]. To date, our research group has conducted considerable research on PT [21,22,23]; for example, we grafted triethylenetetramine on polyacrylonitrile fiber, and then we immobilized persimmon tannin as a bio-sorbent to successfully remove Au (III) from the mixed solution. During the adsorption process, the adsorbent hardly adsorbed metal ions such as Ni(II), Zn(II), and Cu(II) in the solution, which further confirmed that persimmon tannin has selective adsorption to some metal ions [23]. However, in these papers, there are few related mechanism studies on the separation of indium and iron ions, especially theoretical simulation.

In recent years, density functional theory (DFT) has been widely used in organic systems [24]. Furthermore, this method is suitable for determining the feasibility of metal compounds. The EGCG monomer in the PT structure controls the main adsorption function. As a result, the relationship between EGCG and metal ions becomes a bridge to exploring the adsorption mechanism of PT using DFT. Jiang et al. studied the interaction between EGCG and Zn(II) through experiments and theoretical calculations and determined the reaction sites in EGCG for the adsorption of Zn(II) [25]. Our research group used DFT to study the interaction of EGCG with several cations and found that adsorption mainly occurs through three mechanisms: chelation, electrostatic attraction, and the aromatic ring stacking effect [26]. Up to now, theoretical model research on the selective adsorption of indium and iron and the related adsorption mechanisms has not yet been reported.

Herein, this study aims to investigate the selective adsorption mechanism of EGCG with indium and iron ions in their mixture solution in theoretical simulation. The interaction of EGCG with iron and indium ions was studied through DFT calculations by analyzing the bond length, bond order, and binding energy of EGCG–Fe(III) and EGCG–In(III) units, as well as by conducting reduced density gradient (RDG) and atoms-in-molecules (AIM) analyses. Finally, the feasibility of EGCG in the separation of indium and iron ions was further proved by adsorption experiments.

## 2. Materials and Methods

### 2.1. Computational Methods

In this study, the DFT package in Gaussian09-D.01 software was used. Considering the influence of the weak effect, the B3LYP-D3 function method [27,28] was used to calculate the adsorption model of EGCG for indium and iron ions. Mixed basis sets were used in the calculation, i.e., for C, H, and O atoms; the 6-311G (d,p) basis set was used to optimize the structure; the more accurate 6-311+G (d,p) basis set was used to calculate the single point; the Lanl2dz pseudopotential basis set was used for the In and Fe atoms. To simulate the environment in the solution, the SMD solvation model was added to the calculation, and water was used as the solvent. Considering that EGCG is in weakly acidic or neutral solutions, a deprotonation reaction may occur [29]. Hence, the adsorption models of deprotonated and un-deprotonated EGCG were studied in the calculation. All geometric structures in this study were inferred by comparison with the characteristic frequencies and confirmed as the minimum local energy points. The binding energy (Δ*E*) equation to determine the strength of the binding capacity between the adsorbent and the cations is as follows [30]:

(1) $$\Delta E=E_{a}+E_{b}-E_{ab}$$

where *E_a_* and *E_b_* are the energies of the reactants, and *E_ab_* is the energy of the product.

### 2.2. Batch Adsorption Experiment

Twenty milliliters of each of 5 × 10^−3^ mol/L InCl_3_ and 5 × 10^−3^ mol/L FeCl_3_ solutions were added to six clean Erlenmeyer flasks and mixed well, following which 20 mL of 3 × 10^−3^ mol/L EGCG solution was added to each mixture. HCl and NaOH solutions were used to adjust the pH of the solutions in the Erlenmeyer flasks to 1.0, 1.5, 2.0, 2.5, and 3.0 while maintaining one flask as a reference/blank. The maximum pH level was adjusted to below 3.0 because iron ions started to precipitate when the pH exceeded 3.0. The effect of adsorbent dosage was carried out by taking a 1:1 mixed solution with varying masses of the EGCG (0.001, 0.002, 0.003, 0.004, 0.005 mol/L) at pH 3.0. The mixed solution was allowed to react at 25 °C for 6 h for the adsorption to occur. Kinetic studies were carried out by using 0.004 mol/L of EGCG at three different In(III) and Fe(III) initial concentrations of 0.001, 0.003, and 0.006 mol/L and agitated for the fixed time intervals. Adsorption thermodynamic studies were carried out at three temperatures of 303, 308, and 313 K. Thereafter, the supernatant was removed, and the concentration of indium and iron ions in each solution was determined using an atomic absorption spectrometer (Zeenit700P, Analytik Jena AG, Jena, Germany). The adsorption rate was calculated according to the following equation:

(2) $$\mathrm{Adsorption}\;\mathrm{rate}\;\left(\%\right)=\frac{C_{0}-C_{e}}{C_{0}}\times 100\%$$

where *C*_0_ and *C_e_* are the concentrations of one of the metal ions in the mixed solution before and after the reaction, respectively.

### 2.3. The Pseudo-First- and Pseudo-Second-Order Models

The kinetics data were analyzed using pseudo-first-order and pseudo-second-order models. Note that the pseudo-first-order kinetic model assumes that the rate of change of solute uptake with time is directly proportional to the difference in saturation concentrations. By comparison, the pseudo-second-order kinetic model considers that the adsorption process is controlled by the surface reaction. The pseudo-first- and pseudo-second-order models are represented by Equations (3) and (4).

(3) $$\ln\left(q_{e}-q_{t}\right)=\ln q_{e}-k_{1}t$$

(4) $$\frac{t}{q_{t}}=\frac{1}{k_{2}q_{e}^{2}}+\frac{1}{q_{t}}t$$

where *q_t_* (mg·g^−1^) and *q_e_* (mg·g^−1^) are the adsorption capacities of In(III) ions at any time *t* and equilibrium, respectively; and *k*_1_ (min^−1^) and *k*_2_ (min^−1^·g·mg^−1^) represent the rate constants of the pseudo-first-order and pseudo-second-order models, respectively.

### 2.4. The Langmuir and Freundlich Models

The relationship between the amount of In(III) in the left liquid phase and that adsorbed at equilibrium can be represented by an adsorption isotherm. The experimental adsorption isotherm data were fitted by the Langmuir and Freundlich models (Equations (5) and (6)).

(5) $$q_{e}=\frac{K_{L}q_{m}c_{e}}{1+K_{L}c_{e}}$$

(6) qe=Kfce1n

where *q_e_* corresponds to the amount of In(III) ions adsorbed per gram of adsorbent (mg·g^−1^) and *c_e_* represents the equilibrium concentration of In(III) ions in the solution. *q_m_* and *K_L_* denote the Langmuir constants related to the maximum adsorption capacity (mg·g^−1^) and adsorption energy (L·g^−1^), respectively, *K_f_* and (1/*n*) are rough indicators of the adsorption of adsorption intensity, respectively.

### 2.5. Thermodynamic Analysis

The calculated thermodynamic parameters, such as the Gibbs free energy change (Δ*G*^0^), the enthalpy change (Δ*H*^0^) and the entropy change (Δ*S*^0^) can be used to explore the thermal adsorption of In(III) on EGCG as a function of temperature. The thermodynamic parameters were calculated by Equations (7)–(9).

(7) $$\mathrm{Gibbs}\;\mathrm{free}\;\mathrm{energy}:\;lnK_{C}=\frac{\Delta S^{0}}{R}-\frac{\Delta H^{0}}{RT}$$

(8) $$\mathrm{Enthalpy}\;\mathrm{change}:\;\Delta G^{0}=-RTlnK_{C}$$

(9) $$\mathrm{Entropy}\;\mathrm{change}:\;K_{C}=\frac{C_{0}}{C_{e}}$$

### 2.6. Materials and Characterization

EGCG, indium trichloride (InCl_3_), and iron trichloride (FeCl_3_) were procured from Shanghai Aladdin Biochemical Technology Co., Ltd., Shanghai, China. All other chemicals were of analytical grade and used without further purification. The price for direct purchase of epigallocatechin gallate is ¥96/100 mg, while the raw material persimmon tannin is nearly free.

Field emission scanning electron microscopy (FE-SEM, Quanta 450, FEI, Hillsboro, OR, USA) was used to observe the morphology of EGCG before and after adsorption. Simultaneously, the energy-dispersive X-ray spectrometer (EDS) was used to analyze the distribution and proportion of each element on the surface of EGCG in combination with FE-SEM. An X-ray photoelectron spectrometer (XPS, ESCALAB 250Xi, Thermo Fisher, Waltham, MA, USA) was used to analyze the elemental composition of EGCG after the adsorption of In(III)/Fe(III). The BET surface area, pore size, and pore volume of the EGCG were determined by the Micromeritics instrument (ASAP, 2020, Norcross, GA, USA). The total specific surface areas were calculated by the Brunauer–Emmett–Teller (BET) standard equation, and the total pore volumes were calculated at P/P_0_ ≈ 0.99 using the single-point adsorption model. Thermogravimetric analysis (STA 449 F3, Netzsch Company, Selb, Germany) was carried out at a heating rate of 10 °C/min under air between 50 and 800 °C. The X-ray diffraction (XRD) patterns were performed on an Empyrean PIXcel3D (PANalytical, Almelo, The Netherlands) X-ray diffractometer with Cu Kα radiation of λ = 1.5405 Å within the 2θ range of 10°–80°.

## 3. Results and Discussion

### 3.1. Construction of the EGCG–Cation Complex

**Figure 2:**
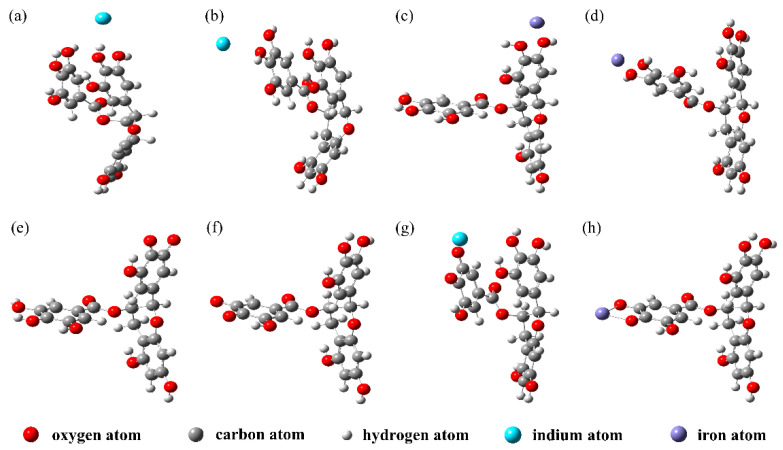
Structural models of EGCG–metal complexes, namely, (**a**) B-EGCG–In(III), (**b**) D-EGCG–In(III), (**c**) B-EGCG–Fe(III), and (**d**) D-EGCG–Fe(III), and deprotonated EGCG–metal complexes, namely, (**e**) B(-2H)-EGCG, (**f**) D(-2H)-EGCG, (**g**) D(-2H)-EGCG–In(III), and (**h**) D(-2H)-EGCG–Fe(III).

Figure 1b shows the structure of EGCG. The four six-membered rings of EGCG are identified as A, B, C, and D. Since EGCG is relatively stable in strong acid solutions [29], its reaction with cations mainly occurs on two pyrogallols, which are the 3′ and 4′ phenolic hydroxyl groups of the B ring and the 4″ and 5″ phenolic hydroxyl groups of the D ring [26,31]. Based on these two active sites, we constructed and optimized EGCG–Fe(III) and EGCG–In(III)) complex models, as shown in Figure 2a–d. The B ring of EGCG reacted with In(III), and the product was defined as B-EGCG–In(III), while the D ring of EGCG reacted with In(III), and the product was defined as D-EGCG–In(III). A similar nomenclature was used for the reactions of Fe(III) at the B and D rings of EGCG.

DFT calculations showed that the free energy of the B-EGCG–In(III) complex was −1678.617 Hartree, lower than that of D-EGCG–In(III) (−1678.615 Hartree). The free energy of B-EGCG–Fe(III) was −1800.271 Hartree, lower than that of D-EGCG–Fe(III) (−1800.199 Hartree). Since low energy indicates high stability, B-EGCG–In(III) (Figure 2a) and B-EGCG–Fe(III) (Figure 2c) were selected as the subsequent calculation models for the adsorption of In(III) and Fe(III) on EGCG, respectively.

Since EGCG readily undergoes a significant deprotonation reaction with increasing solution pH [29], the B and D ring models of deprotonated EGCG were constructed, and their geometric structures were optimized (Figure 2e,f). Comparing the single point energies, it was found that the free energy of D(-2H)-EGCG was −1676.308 Hartree, lower than that of B(-2H)-EGCG (−1676.298 Hartree); thus, D(-2H)-EGCG (Figure 2f) was selected as the EGCG model for subsequent deprotonation. The deprotonated EGCG was then used to construct complex models with indium and iron cations and optimize the structure (Figure 2g,h).

### 3.2. Bond Length and Bond Order of EGCG–Metal Complexes

The bond length and bond order are important parameters for determining the bond strength of EGCG with metal ions. In this study, the Multiwfn program [32] was used to investigate the bond length and Mayer bond order [33] of the complexes. The calculation results are listed in Table 1. When the solution was strongly acidic, the binding models of EGCG with indium and iron ions were B-EGCG–In(III) and B-EGCG–Fe(III), respectively. By comparing the parameters, we found that the bond length of B-EGCG–Fe(III) was much shorter than that of B-EGCG–In(III), whereas the Mayer bond order of B-EGCG–Fe(III) was greater than that of B-EGCG–In(III); this implies that B-EGCG readily adsorbed Fe(III) over In(III) and had higher binding strength for Fe(III) than that for In(III). When the solution was weakly acidic or neutral, the models of EGCG combined with indium and iron ions were D(-2H)-EGCG–In(III) and D(-2H)-EGCG–Fe(III), respectively. Furthermore, it was found that D(-2H)-EGCG, similar to B-EGCG, exhibited better adsorption for Fe(III) than that for In(III), and that the formation of complexes was easier. The Mayer bond order of D(-2H)-EGCG and B-EGCG with In(III) was close to 0, indicating the absence of chelation between In(III) and the two monomers; in contrast, the Mayer bond order of D(-2H)-EGCG and B-EGCG with Fe(III) was in the range of approximately 0.21–0.36, implying that the adsorption of Fe(III) resulted in the formation of weak chelating bonds in the complex.

### 3.3. RDG Analysis

Using RDG function analysis, the type of weak interaction between atoms can be determined by drawing isosurfaces of different colors [34]. The RDG function is a real space function describing the deviation of uniform electron distribution, given by [26]:

(10) $$RDG\left(r\right)=\frac{1}{2{\left(3\pi^{2}\right)}^{1/3}}\frac{\left|\nabla \rho \left(r\right)\right|}{\rho {\left(r\right)}^{4/3}}$$

where ∇ is the gradient operator, and |∇*ρ*(*r*)| is the modulus of the electron density gradient. The weak interaction is represented by the gradient isosurface formed in the corresponding region in three-dimensional space.

**Figure 3:**
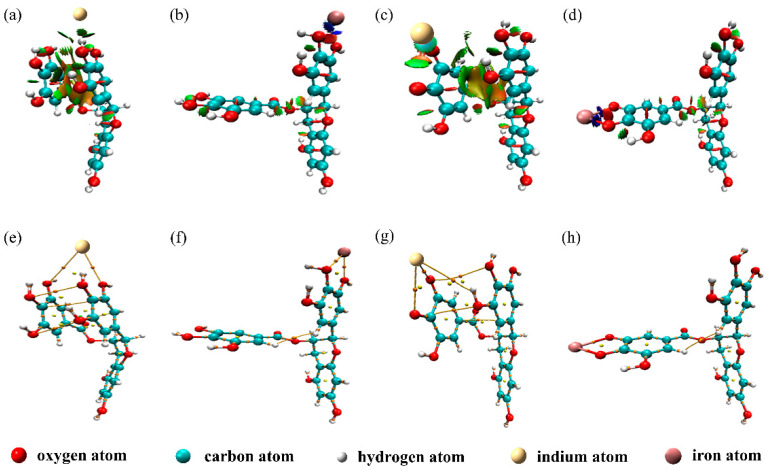
RDG function isosurface map of EGCG–metal composites: (**a**) B-EGCG–In(III), (**b**) B-EGCG–Fe(III), (**c**) D(-2H)-EGCG–In(III), and (**d**) D(-2H)-EGCG–Fe(III). AIM analysis profiles of EGCG–metal complexes: (**e**) B-EGCG–In(III), (**f**) B-EGCG–Fe(III), (**g**) D(-2H)-EGCG–In(III), and (**h**) D(-2H)-EGCG–Fe(III).

To determine the presence of the weak interaction between EGCG and indium/iron ions, we imported the optimized model file into the Multiwfn software and combined it with the VMD software [35] to draw the RDG function isosurface map of the EGCG–metal composite, as shown in Figure 3a–d. The blue isosurface in the figure indicates that the interaction is strong, and that the contenders are hydrogen and halogen bonds. The green area indicates that the interaction is weak, which corresponds to the van der Waals effect. The red area indicates strong mutual repulsion, which could be the strong steric hindrance in the ring or the chelate cage.

By comparing Figure 3a,c, we can infer that In(III) formed the green isosurface with B-EGCG and D(-2H)-EGCG. When In(III) was adsorbed, the structures of B-EGCG and D(-2H)-EGCG underwent major changes (the D ring folded and approached the B ring); this resulted in the formation of a large area of yellow–green isosurface between the B and D rings of EGCG in the RDG analysis, which confirmed the presence of the aromatic ring stacking effect in adsorption. In Figure 3b, blue isosurfaces between Fe(III) and the 3′O and 4′O of B-EGCG can be observed, indicating a strong attraction between the two through electrostatic interactions. However, Figure 3d highlights another observation—there are not only blue isosurfaces between Fe(III) and the 4″O and 5″O of D(-2H)-EGCG but also chelating bonds, which indicates that D(-2H)-EGCG has electrostatic and chelation effects in the adsorption of Fe(III). This also confirmed to a certain extent that the adsorption between Fe(III) and D(-2H)-EGCG is much stronger than that between Fe(III) and B-EGCG.

### 3.4. AIM Analysis

AIM analysis [36] is currently one of the most popular wave function analysis methods and is often used to analyze the chemical bonds between atoms. In AIM analysis, the most common discussion is about the critical point of the electron density function, which is the point where the modulus of the function gradient is 0. The bond critical point (BCP) corresponds to the second-order saddle point of the function, which exists between the interacting atoms. To investigate the interaction between EGCG and indium/iron ions, both Multiwfn and VMD were used to draw the AIM topology analysis diagram, as shown in Figure 3e–h. The orange point in the figure is the BCP under consideration, and the yellow line between atoms is the bond diameter. BCPs between indium/iron ions and EGCG–related interaction sites can also be seen in Figure 3e–h. From the observation of Figure 3e,g, we found that when In(III) was adsorbed, BCPs also appeared between the B and D rings of EGCG, which proved that there was an interaction between these two rings. Notably, in Figure 3h, Fe(III) and D(-2H)-EGCG’s 4″O and 5″O not only had yellow bond diameters but also stable chelate bonds, BCPs were located in the middle of the chelate bonds, which was proved that the complex of deprotonated EGCG and Fe(III) was relatively stable, and there was certain chelation between the two.

**Figure 4:**
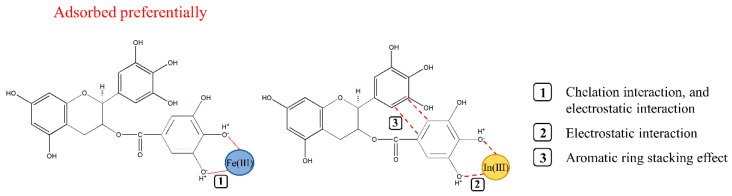
Diagram of the possible interaction mechanism between the deprotonated EGCG and Fe(III)/In(III).

The electron density of the BCP position is closely related to the strength of the chemical bond. Table 2 lists the calculated electron density ρ(BCP) of some BCP positions in the complexes. Typically, the comparison of ρ(BCP) is limited to the chemical bond between two specific elements. The larger the ρ(BCP), the stronger the chemical bond. Table 2 shows that the values of ρ(BCP) between D(-2H)-EGCG and In(III) were 0.01464 and 0.02335 a.u. for the 4″O and 5″O positions, respectively; these values were greater than those of B-EGCG–In(III) (0 and 0.00917 a.u. for the 3′O and 4′O positions, respectively). Similarly, the values of ρ(BCP) between D(-2H)-EGCG and Fe(III) were greater than those between B-EGCG and Fe(III). This further confirms that deprotonated EGCG has higher binding strength for indium/iron ions than does B-EGCG. Combining the two weak interaction analysis methods, RDG and AIM, we speculated on the interaction of deprotonated EGCG with Fe(III)/In(III), as shown in Figure 4.

### 3.5. Binding Energy between EGCG and Indium/Iron Ions

**Figure 5:**
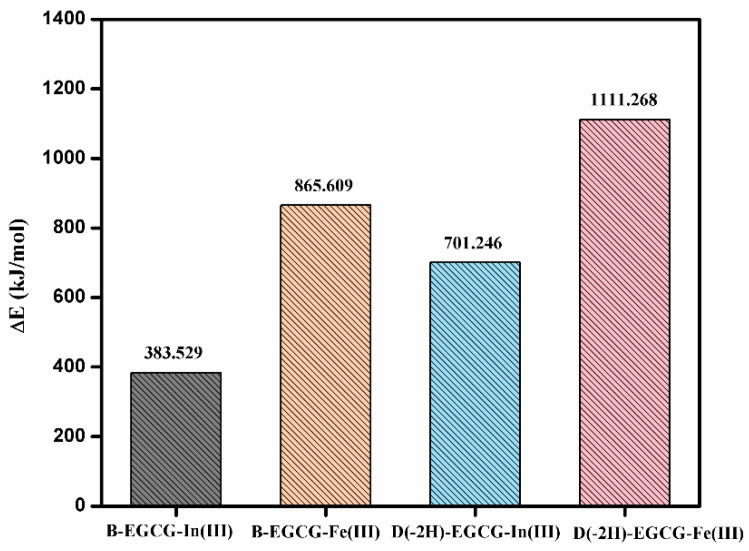
The binding energy of EGCG–metal complexes.

The binding energy between EGCG and metal ions determines the stability of the composite. Figure 5 shows the calculated binding energy between EGCG and indium/iron ions, wherein the binding energy of B-EGCG for In(III) is 383.5 kJ/mol, significantly lower than that for Fe(III) (865.6 kJ/mol). The binding energy of D(-2H)-EGCG for In(III) is 701.2 kJ/mol, lower than that for Fe(III) (1111.3 kJ/mol). This confirms that the ability of EGCG for adsorbing Fe(III) in acidic or neutral solutions was always greater than that for absorbing In(III), i.e., EGCG preferentially adsorbed iron ions in indium–iron solutions. For the same metal ion, Fe(III) or In(III), the bonding strength between the ion and D(-2H)-EGCG was also better than that between the ion and B-EGCG.

### 3.6. Characterization of the EGCG

**Figure 6:**
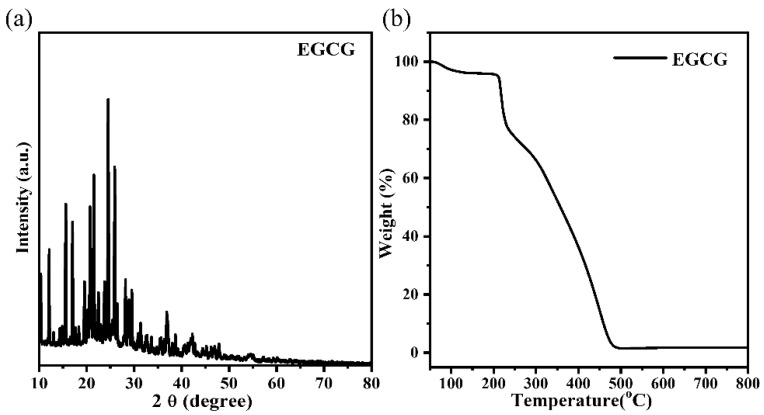
(**a**) XRD patterns of EGCG; (**b**) TGA analysis of EGCG.

XRD was used to evaluate the crystal structural phase of the EGCG sample. As seen in Figure 6a, pure EGCG had numerous characteristic peaks at 2θ of 10.35°, 12.12°, 15.61°, 21.54°, 24.54°, 25.93°, etc., suggesting that it was the EGCG crystalline structure [37]. The thermogravimetric (TGA) analysis of the EGCG was investigated and is shown in Figure 6b. The thermogram of EGCG presents a two-step mass loss, which is attributed to the loss of water (<120 °C) and thermal degradation (>218 °C) [38]. The specific surface area and pore size of the adsorbent are factors that affect the adsorption performance. The specific surface area, average pore diameter, and total pore volume of EGCG in this work were 2.67 m^2^/g, 14.21 nm, and 0.0095 cm^3^/g, respectively. As shown in Figure S1, the EGCG exhibited a typical type-II isotherm, which rose gradually as the relative pressure increased. The BET parameters and isotherm both indicated the EGCG was not a porous material.

### 3.7. Adsorption Kinetics Studies

**Figure 7:**
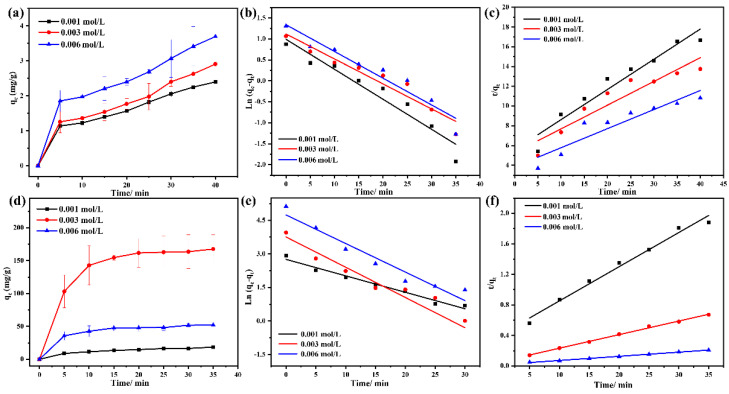
(**a**) The effect of the different initial In(III) concentration on the adsorption capacity; the linear fit with pseudo−first−order (**b**) and pseudo−second−order (**c**) models of In(III) adsorbed on EGCG. (**d**) The effect of the different initial Fe(III) concentrations on the adsorption capacity; the linear fit with pseudo−first−order (**e**) and pseudo−second−order (**f**) models of Fe(III) adsorbed on EGCG.

For the adsorption kinetic studies, the effects of contact time on the adsorption capacity at different In(III) or Fe(III) initial concentrations are outlined in Figure 7. At different In(III) or Fe(III) concentrations, the adsorption capacity gradually increased with increasing contact time (Figure 7a,d). Two kinetic models were used to fit the experimental data of In(III) (Figure 7b,c) and Fe(III) (Figure 7e,f) in the work. The fitting results of the kinetic parameters are listed in Tables S1 and S2. Seen from Table S1, the value of the correlation coefficient R^2^ fitted by the pseudo-first-order kinetic model was closer to 1, indicating that the pseudo-first-order model was more suitable for describing In(III) adsorbed on EGCG. However, the fitting kinetic parameters of Fe(III) adsorption data are presented in Table S2. The correlation coefficient R^2^ of the pseudo-second-order kinetic model was higher than that of the pseudo-first-order kinetic model and closer to 1. As such, the pseudo-second-order kinetic model was considered to be the most suitable for describing the adsorption behavior of Fe(III) adsorbed on EGCG. It also suggested that the adsorption behavior of Fe(III) was mainly controlled by chemisorption [39].

### 3.8. Adsorption Isotherm Studies and Thermodynamics Analysis

**Figure 8:**
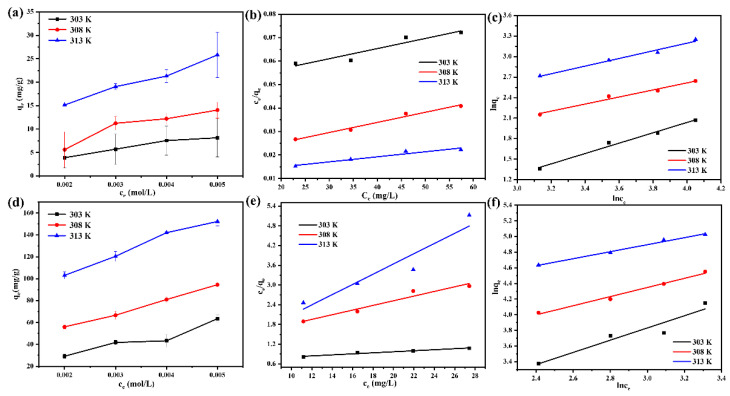
(**a**) The effect of initial concentration on the adsorption capacity; the fitting results of the experimental adsorption isotherm data in terms of Langmuir (**b**) and Freundlich (**c**) models of In(III) adsorbed on EGCG. (**d**) The effect of initial concentration on the adsorption capacity; the fitting results of the experimental adsorption isotherm data in terms of Langmuir (**e**) and Freundlich (**f**) models of Fe(III) adsorbed on EGCG.

The adsorption process of the EGCG was evaluated by fitting the adsorption isotherm equation with the experimental results. As shown in Figure 8a,d, the adsorption capacity increased with the increase of In(III) or Fe(III) concentrations and temperature. The Langmuir model and the Freundlich model were used to evaluate the adsorption process of EGCG for In(III) and Fe(III). The fitted model curves are shown in Figure 8b,c,e,f, and the fitted parameters are shown in Tables S3 and S4. According to the obtained data, we found that the R^2^ of the Freundlich isotherm model was closer to 1 than that of the Langmuir model. Thereby, the Freundlich model was a better fit for characterizing the In(III) and Fe(III) adsorption isotherms. These results suggest that the adsorption of In(III) and Fe(III) onto the EGCG surface followed a multi-site adsorption mechanism for heterogeneous surfaces.

In order to study the nature of the adsorption process. we calculated the Gibbs free energy change (Δ*G*_0_), enthalpy change (Δ*H*_0_), and entropy change (Δ*S*_0_) from a thermodynamic point of view. As shown in Tables S5 and S6, for In(III) and Fe(III) adsorption on EGCG, the negative value Δ*G*_0_ implied the spontaneous nature of the process. The positive value of Δ*H*_0_ indicated that In(III) or Fe(III) was adsorbed as an endothermic process. Moreover, it has been reported that if adsorption Δ*H*_0_ ranges from 20.9 to 418.4 kJ/mol, which is similar to the heat of chemical reactions, adsorption is often assumed to be associated with chemisorption, and the value Δ*H*_0_ of Fe(III) further implies that the nature of Fe(III) adsorption on EGCG is endothermic chemisorption [20].

### 3.9. Selective Adsorption Experiments of Indium and Iron Ions

**Figure 9:**
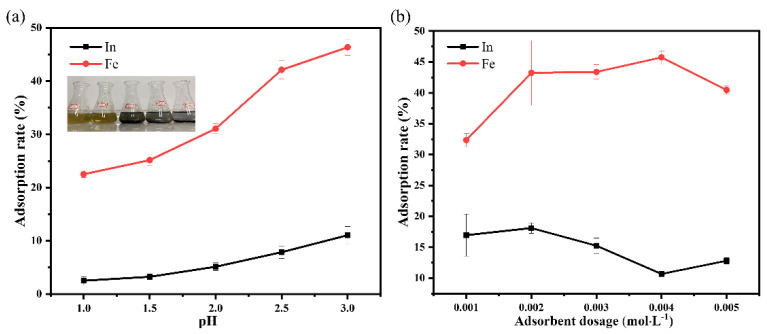
Adsorption rates of EGCG with indium and iron ions in solutions with different (**a**) pH values and (**b**) adsorbent dosages.

The selective adsorption capacity of EGCG for Fe(III) and In(III) was further verified using the designed tests. The adsorption rates of EGCG in solutions with different pH values are shown in Figure 9a. It was found that with more blue–purple precipitates, which were produced by the adsorption of Fe(III) and EGCG and increased with increasing pH in the mixed solution (1:1 molar ratio), the solution became increasingly colorless and transparent after the adsorption reaction. Figure 9a shows that when EGCG was used as an adsorbent in the mixed solution of indium and iron, the adsorption rate of In(III) on EGCG was low, approximately 2.5% to 11.1%; by contrast, the adsorption rate of Fe(III) was high and increased gradually with increases in pH. This indicates that the adsorptions of In(III) and Fe(III) were competitive processes, and that the adsorption of Fe(III) on EGCG was always better than that of In(III). When the pH level of the mixed solution reached 3.0, the adsorption capacity of EGCG reached a maximum (iron ions started to precipitate when the pH exceeded 3.0). Here, the adsorption rate was 46.4% for Fe(III) and 11.1% for In(III). Typically, at low values of pH, a large number of phenolic hydroxyl groups in EGCG are in an undissociated state, which is not conducive to the adsorption of Fe(III) or In(III). With an increase in the pH of the solution, EGCG begins to dissociate, and the deprotonated EGCG forms its anionic group, which is conducive to the adsorption of cations. The adsorbent dose is another important parameter because it determines the capacity of a sorbent for a given initial concentration. The effect of the adsorbent dose was studied by varying the adsorbent in the range of 0.001–0.005 mol/L at pH 3.0 in the 1:1 mixed solutions, and the results are shown in Figure 9b. As seen in Figure 9b, when the adsorbent dosage increased from 0.001 to 0.005 mol/L, the adsorption rate of Fe(III) gradually increased to 45.7%, while the adsorption rate of In(III) gradually decreased to 10.7%. Therefore, the optimal EGCG adsorbent dose was 0.004 mol/L. Our calculation results in both the deprotonated and un-deprotonated cases also supported this observation. Moreover, the method usually used to separate indium and iron is to directly adsorb indium ions in wastewater and then desorb indium ions with eluate to obtain a solution rich in indium [40,41,42]. However, in our study, we enriched indium ions in wastewater solution by selectively adsorbing unwanted iron ions, thus simplifying the secondary desorption step and contributing to actual production.

### 3.10. SEM Characterization of Indium/Iron Ion Adsorption by EGCG

For the solution with a pH of 3.0, the SEM images of EGCG before and after adsorption (Figure S2a,b) showed that the adsorbent before the adsorption had a lumpy structure with an irregular surface. These irregular structures provided more active sites for the adsorption of metal ions. The materials were analyzed via EDS surface scans before and after the adsorption; the changes in the elemental contents before and after the adsorption are shown in Figure S2c,d. It was found that both indium and iron were present in the adsorbed precipitate, which indicates that EGCG could successfully adsorb Fe(III) and In(III). At the same time, the atomic percentage of iron in the adsorbed material (1.98%) was much higher than that of indium; this additionally confirms that EGCG can selectively adsorb Fe(III) in an indium–iron mixed solution. Moreover, the EDS mapping results (Figure S2e–h) showed that the C, O, Fe, and In elements were homogeneously distributed throughout the adsorbent surface, indicating that the binding sites of Fe and In ions and adsorbent were evenly distributed in the adsorbent material.

### 3.11. XPS Analysis of the Indium/Iron Ion Adsorption by EGCG

XPS was used to analyze the elemental composition of EGCG after the adsorption of indium/iron ions in the solution with a pH of 3.0. The results are shown in Figure S3. We can see that the electron orbital peaks of C 1s, In 3d, O 1s, and Fe 2p appeared at 284.18, 444.98, 532.38, and 709.98 eV, respectively. This shows that the adsorbed complex was mainly composed of C, In, O, and Fe. The contents of these elements were 67.34%, 0.62%, 30.61%, and 1.44%, respectively, which are similar to those obtained with the EDS analysis after the EGCG adsorption of indium/iron ions. The XPS results confirmed that EGCG successfully adsorbed Fe(III) and In(III) in the mixed solution, and that it was significantly selective to the adsorption of Fe(III) in the solution with a pH of 4.0.

## 4. Conclusions

Computational analysis based on the DFT method was used to investigate the selective adsorption mechanism of adopting EGCG in indium–iron separation. The DFT results indicated that the bonding strengths of deprotonated EGCG–[Fe(III) or In(III)] complexes were higher than those of un-deprotonated complexes, and the deprotonated EGCG was easier to combine with indium/iron cations. RDG analysis confirmed that the interaction of the EGCG–Fe(III) complex was dominated by chelation and electrostatic interactions, while that of the EGCG–In(III) complex was controlled by electrostatic interactions and the aromatic ring stacking effect. Fe(III) exhibited a higher binding capacity with EGCG than that with In(III) in acidic or neutral solutions by the calculation of binding energy. Finally, the adsorption experiment confirmed that the Freundlich equilibrium isotherm fit the In(III) and Fe(III) adsorption behavior very well, and the Fe(III) adsorption processes followed the pseudo-second-order model. Thermodynamics data revealed that the adsorption of In(III) and Fe(III) onto EGCG was feasible, spontaneous, and endothermic. The EGCG monomer had a stronger affinity for Fe(III) than that for In(III), consistent with theoretical model predictions. In the 1:1 mixed solution with a pH of 3.0, the adsorption rate of Fe(III) on EGCG was 45.7%, which significantly exceeded that of In(III) (10.7%). These results provided further insights into the design and performance optimization of related tannin-based adsorbents from the perspective of the molecular level.

## Figures and Tables

**Table 1:** Bond length and bond order of EGCG–metal complexes.

EGCG–Metal Complex	Compound Bond	Bond Length (Å)	Mayer Bond Order
B-EGCG–In(III)	3′O-In	3.1418	0.0425
	4′O-In	4.0454	0.0188
B-EGCG–Fe(III)	3′O-Fe	2.1682	0.2211
	4′O-Fe	2.2139	0.2193
D(-2H)-EGCG–In(III)	4″O-In	2.9289	0.0742
	5″O-In	2.6831	0.1183
D(-2H)-EGCG–Fe(III)	4″O-Fe	2.0819	0.3321
	5″O-Fe	2.0721	0.3623

**Table 2:** Electron density at different BCP positions in the complexes.

EGCG–Metal Complex	Compound Bond	ρ(BCP) (a.u.)
B-EGCG–In(III)	3′O-In	0.00917
	4′O-In	0
B-EGCG–Fe(III)	3′O-Fe	0.04504
	4′O-Fe	0.04102
D(-2H)-EGCG–In(III)	4″O-In	0.01464
	5″O-In	0.02335
D(-2H)-EGCG–Fe(III)	4″O-Fe	0.06035
	5″O-Fe	0.06289

## Data Availability

The data presented in this study are available upon request from the corresponding author.

## Supplementary Materials

The following supporting information can be downloaded at: https://www.mdpi.com/article/10.3390/ma15228251/s1, Figure S1: Nitrogen adsorption-desorption isotherms of the EGCG; Figure S2: (a) SEM image of EGCG before adsorption; (b) SEM image of EGCG after adsorption; (c) EDS image of EGCG before adsorption; and (d) EDS image of EGCG after adsorption; (e–h) EDS mapping images of EGCG after adsorption; Figure S3: XPS spectrum of EGCG after indium/iron ion adsorption; Table S1: Kinetic parameters for the adsorption of In(III) by EGCG at different concentrations; Table S2: Kinetic parameters for the adsorption of Fe(III) by EGCG at different concentrations; Table S3: Isotherm parameters for adsorption of In(III) by EGCG at different temperatures; Table S4: Isotherm parameters for adsorption of Fe(III) by EGCG at different temperatures; Table S5: Thermodynamic parameters for adsorption of In(III); Table S6: Thermodynamic parameters for adsorption of Fe(III).
