# Supplementary material for: Computational and Experimental Investigation of the Selective Adsorption of Indium/Iron Ions by the Epigallocatechin Gallate Monomer
Source: Materials (Basel). 2022 Nov 21;15(22):8251. doi: 10.3390/ma15228251 (PMC9696512; doi:10.3390/ma15228251)
Supplement: Supplementary file 1 [file materials-15-08251-s001.zip › materials-1951053-supplementary.pdf]

Supporting Information

Computational and Experimental Investigation of the Selective Adsorption of Indium/Iron Ions by the Epigallocatechin Gallate Monomer

Table S1. Kinetic parameters for the adsorption of In(III) by EGCG at different concentrations.

Kinetic Models	Parameters	Concentrations (mg/L)		
		**0.001**	**0.003**	**0.006**
Pseudo-first order	q_e (mg/g)	2.697	3.293	3.817
	k_1	0.0715	0.0596	0.0637
	R^2	0.924	0.925	0.921
Pseudo-second order	q_e (mg/g)	3.27	4.16	5.174
	k_2	0.0168	0.0109	0.00972
	R^2	0.929	0.863	0.857

Table S2. Kinetic parameters for the adsorption of Fe(III) by EGCG at different concentrations.

Kinetic Models	Parameters	Concentrations (mg/L)		
		**0.001**	**0.003**	**0.006**
Pseudo-first order	q_e (mg/g)	15.73	42.78	114.39
	k_1	-0.0734	-0.13514	-0.12757
	R^2	0.970	0.846	0.932
Pseudo-second order	q_e (mg/g)	22.33	56.21	183.15
	k_2	0.0447	0.0177	0.00546
	R^2	0.982	0.997	0.997

Table S3. Isotherm parameters for adsorption of In(III) by EGCG at different temperatures.

Isotherms parameters	Parameters	Temperatures(K)		
		**303**	**308**	**313**
Langmuir	q_{max} (mg/g)	2317	2321	4649
	K_L (L/mg)	0.0089	0.0258	0.0202
	R^2	0.851	0.970	0.915
Freundlich	K_F (mg/g (L/mg)$^{1/n}$)	0.371	1.734	2.612

	1/n	0.75725	0.5163	0.5589
	R^2	0.976	0.970	0.974

Table S4. Isotherm parameters for adsorption of Fe(III) by EGCG at different temperatures.

**Isotherms parameters**	**Parameters**	**Temperatures(K)**		
		**303**	**308**	**313**
Langmuir	q_{max} (mg/g)	86.35	14.122	6.433
	K_L (L/mg)	0.017	0.064	0.291
	R^2	0.935	0.928	0.857
Freundlich	K_F (mg/g (L/mg)$^{1/n}$)	4.423	13.451	34.813
	1/n	0.781	0.583	0.448
	R^2	0.867	0.981	0.987

Table S5. Thermodynamic parameters for adsorption of In(III).

**T(K)**	**ΔG_0(kJ/mol)**	**ΔH_0(kJ/mol·K)**	**ΔS_0(J/mol·K)**
303	-3.3	6.0	28.3
308	-4.9		
313	-4.6		

Table S6. Thermodynamic parameters for adsorption of Fe(III).

**T(K)**	**ΔG_0(kJ/mol)**	**ΔH_0(kJ/mol·K)**	**ΔS_0(J/mol·K)**
303	-3.2	64.3	280.6
308	-4.3		
313	-5.8		

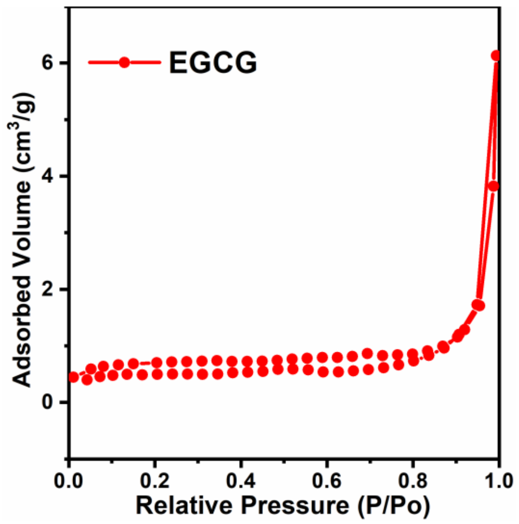

Figure S1. Nitrogen adsorption-desorption isotherms of the EGCG.

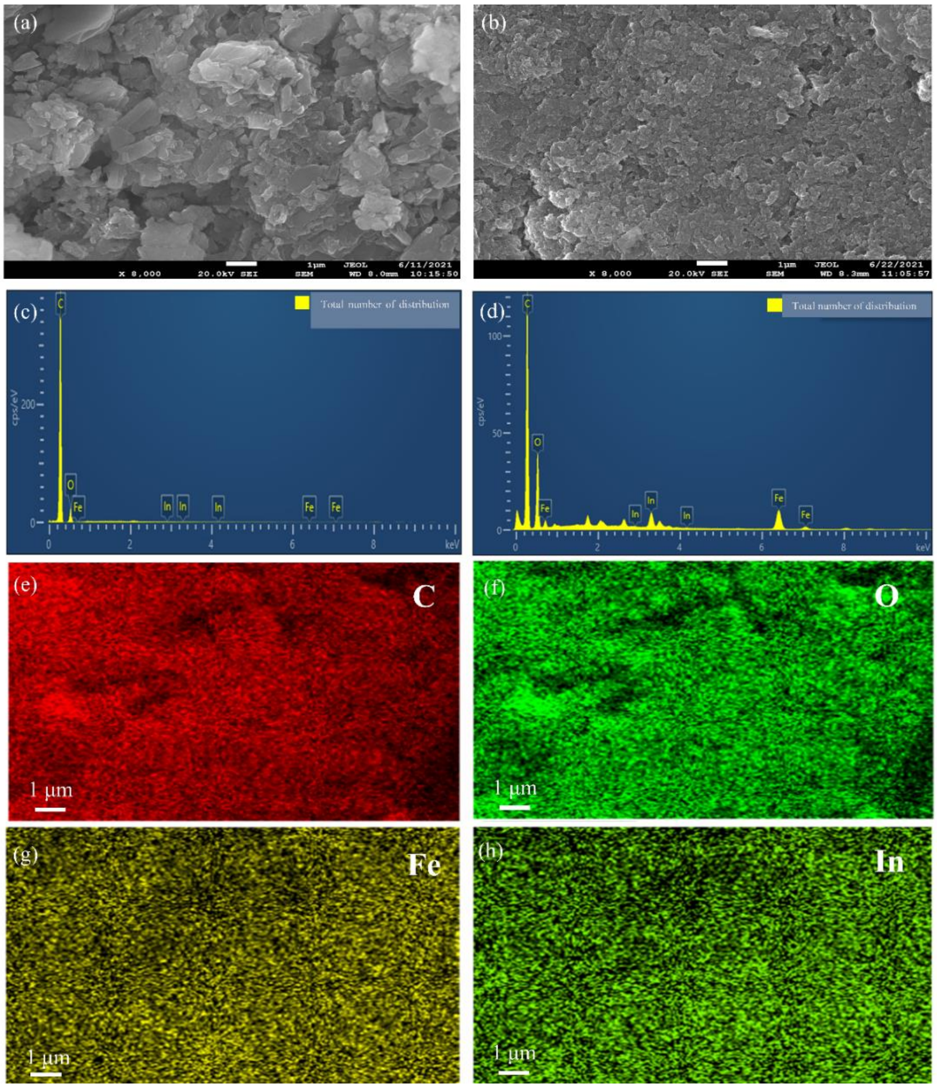

Figure S2. (a) SEM image of EGCG before adsorption; (b) SEM image of EGCG after adsorption; (c) EDS image of EGCG before adsorption; and (d) EDS image of EGCG after adsorption； (e-h) EDS mapping images of EGCG after adsorption.

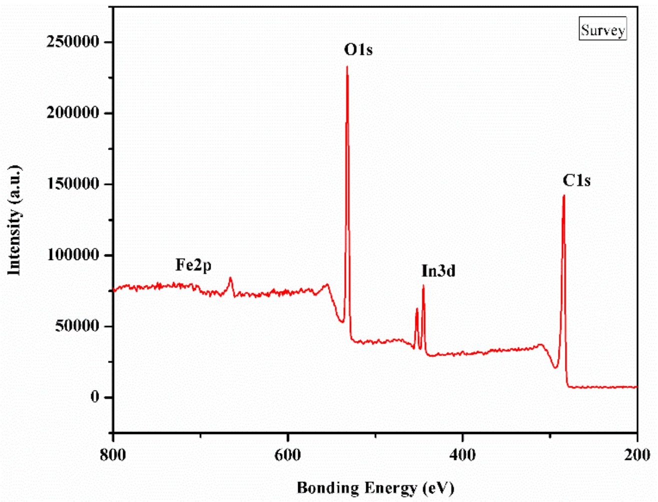

Figure S3. XPS spectrum of EGCG after indium/iron ion adsorption.
